# Field Investigation of Wave Attenuation in a Mangrove Forest Dominated by *Avicennia marina* (Forsk.) Viern.

**DOI:** 10.3390/plants14010135

**Published:** 2025-01-05

**Authors:** Xing Wei, Wenyuan Mo, Lanlan Xiong, Xin Hu, Hao Cheng

**Affiliations:** 1 State Key Laboratory of Tropical Oceanography, South China Sea Institute of Oceanology, Chinese Academy of Sciences, Guangzhou 510301, China; wes@scsio.ac.cn; 2 School of Civil Engineering and Architecture, Hainan University, Haikou 570228, China; redondomo@163.com; 3 Guangdong Center for Marine Development Research, Guangzhou 510220, China; huxinest@163.com

**Keywords:** mangroves, *Avicennia marina*, wave attenuation, drag coefficient, Zhanjiang Bay

## Abstract

Based on field observation at the north coast of the Zhanjiang Bay in southern China, the characteristics of wave attenuation due to the drag force of one mangrove species, *Avicennia marina* (Forsk.) Viern., were quantitatively analyzed. The results demonstrated that the mean significant wave height decreased by ~62% within a forest belt up to 80 m due to various bio-physical interactions. Affected by the unique vertical configuration of vegetation, the wave attenuation rate is positively correlated with water depth. The drag force within the forest can be approximated by the function $C_{d}=0.7344e^{0.1409A_{m}}$, where *A_m_* is the projected area of the submerged obstacle at a certain water depth. The wave attenuation rate and the vegetation density (*ρ_veg_*) in volume (‰) satisfy the fitting relationship of $r=5\times 10^{-4}\cdot \rho_{veg}-3.6\times 10^{-3}$. These findings can accumulate quantitative information for studying the influence of mangrove vegetation on wave attenuation characteristics and provide necessary basic data for modeling studies to investigate the processes contributing to the attenuation capacity of mangroves.

## 1. Introduction

Mangroves are salt-tolerant evergreen woody plant communities in the intertidal zone of tropical and subtropical coastlines. Studies on the function of the wave attenuation of mangroves have been conducted since the late 1990s. These works can be divided into four categories: theoretical [1,2,3], field observations [4,5,6,7], experimental [8,9,10,11] and numerical modeling [12,13,14,15]. Although these studies have their own focus, they all agree that vegetation gives rise to distinct dissipative effects whether inundated or surfaced, rigid or flexible, dense or sparse. However, despite the recognition that mangroves can provide these invaluable ecosystem services, mangrove coverage is still declining rapidly due to a combination of global climate change and human activities [16,17,18]. Therefore, there is an urgent need to explore in depth the physical contribution of mangroves in reducing the vulnerability of coastal areas to hazards such as sea level rise and extreme waves in order to increase awareness of the need for mangrove preservation and adequately optimize the project planning and designing phases for green–grey infrastructure [19,20,21].

Waves propagating through submerged vegetation lose energy due to the turbulent flow separation induced by the stems, roots and branches, resulting in the creation of a drag force. The amount of wave energy dissipated strongly depends on vegetation characteristics including height, density, root structure, etc. [1,2,22,23]. Many studies have attempted to construct a functional relationship between the bulk drag coefficient and wave attenuation rate based on field observation data [5,6,7,24,25]. However, the application of these results to different species must be preceded by a particularly careful comparison and analysis because, as noted by [26], each mangrove species has a unique configuration of trunk, prop roots and pneumatophores that act as different drag forces and consequently lead to different attenuation rates to waves. Unfortunately, the current accumulation of knowledge on the wave attenuation effects of different species of mangroves is far from adequate. This greatly limits the proposal of a better optimal scheme for mangrove-based shelter belt construction, including how to select and breed species, improve stand structure and rationalize the configuration [27]. Accordingly, to effectively enhance the protective effect of mangroves, it is first necessary to accumulate quantitative knowledge of the physical behavior of each mangrove species based on field observations, and then to address the mechanisms associated with wave attenuation for each type of mangrove species.

**Figure 1:**
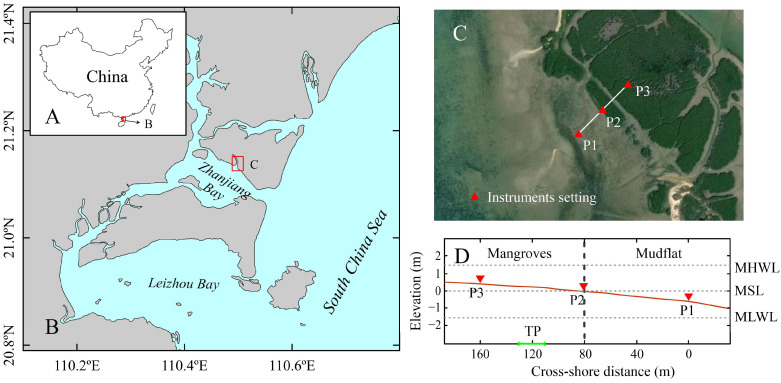
(**A**) Schematic map of the Leizhou Peninsula and its location in relation to China. (**B**) The geomorphology of Zhanjiang Bay and the relative location of the study area. (**C**) Location of hydrological observation sites in the study area. (**D**) Elevation with respect to mean sea level (MSL) of the cross-shore transect including instrument positions. The vegetation zone and mudflat on the transect are demarcated by dashed line. Plot location for the vegetation survey is shown on the horizontal coordinate. Tidal water levels are indicated at the right axis (MHWL, mean highwater level; MLWL, mean low water level).

This paper addresses wave reduction over a tidal flat and within a contiguous *Avicennia marina* (Forsk.) Viern. forest area at the north coast of Zhanjiang Bay in southern China (Figure 1). Field experiments are used to reach the main purpose: to accumulate quantitative information for the wave attenuation effect of *A. marina* and to pursue an explicit relation between the mangrove density and the wave attenuation capacity of mangroves by correlating the wave attenuation with the volume percentage of inundated mangrove biomass. We believe this study will contribute to a better understanding of the role of *A. marina* in wave attenuation, and the valuable site-specific field data presented may also provide evidence and a reference for future coastal protection design, as well as provide basic parameters for further numerical modeling studies.

## 2. Results

### 2.1. Vegetation Traits and Density

**Figure 2:**
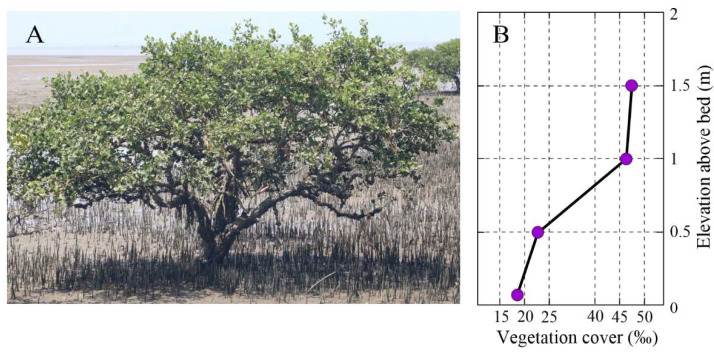
(**A**) Characteristic of *Avicennia marina* (Forsk.) Viern. in the study area. (**B**) Variation in horizontal vegetation cover with elevation above the forest floor.

The measured vegetation distribution density is 0.21–0.43 units/m^2^, gradually increasing along the transect. The plant height is 1.33–2.08 m, and the crown part is 0.44–0.87 m high. The root is surrounded by pneumatophores (6.7–12.3 cm in height and 1.1–2.3 cm in width) with a density range of 88–303 units/m^2^. Figure 2A shows a picture of an adult *A. marina* tree located at the forest fringe surrounded by pneumatophores. The horizontal vegetation coverage in the sampling field increased significantly with increasing elevation above the forest floor (Figure 2B). At 5 cm above the bed, which is the mean height of the pneumatophores, the vegetation coverage is approximately 17‰. Above this layer, the vegetation coverage increases sharply to approximately 47‰ due to the presence of a dense canopy.

### 2.2. Tidal and Wave Climate

**Figure 3:**
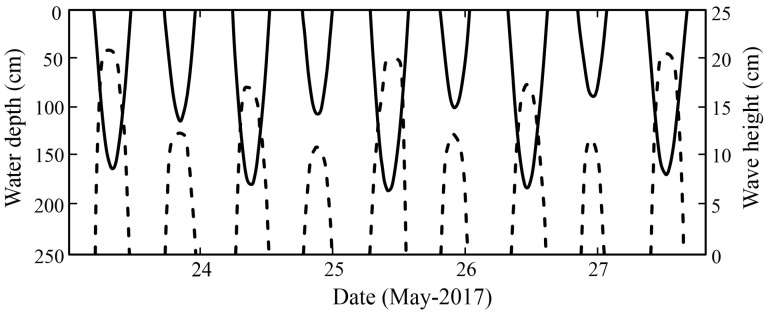
The water depth (solid lines) and wave height (dotted lines) at the outermost station P1 during the observation period.

**Figure 4:**
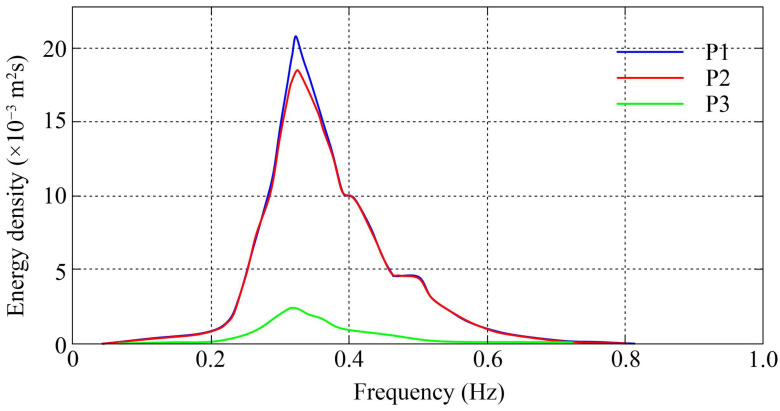
Average wave energy density spectra of wave data during full inundation of the cross-shore transect.

The hydrographic survey recorded the wave variation characteristics of the cross-shore transect for a total of four consecutive tidal cycles during the spring tide. Figure 3 shows the variation in wave and water level at the outermost observation station P1. The highest water depth at P1 reached 1.77 m, and the largest significant wave height was 22.3 cm. However, the correlation between water depth and wave height was not significant, which implies that the effect of depth limitation on incident shallow waves is not significant on the mudflat. Figure 4 shows the time-averaged wave energy density spectra of the observation stations. The wave spectra of all measured stations were mostly uni-modal with wave periods mainly varying between 1.5 and 6.7 s. The peak frequency occurred between 2.5 and 4 s. On the whole, the spectral density showed an obvious decreasing trend in the cross-shore transect. According to [28], wave breaking occurs for wave heights exceeding 60–83% of the water depth, while the observed wave heights on the transect ranged up to only approximately 15% of the simultaneously measured water depths. Therefore, it can be concluded that wave breaking does not contribute significantly to the loss of wave energy, while drag and friction induced by mangrove vegetation and interaction with the forest floor are the main causes of wave attenuation along the transect.

### 2.3. Attenuation Waves over the Mudflats

**Figure 5:**
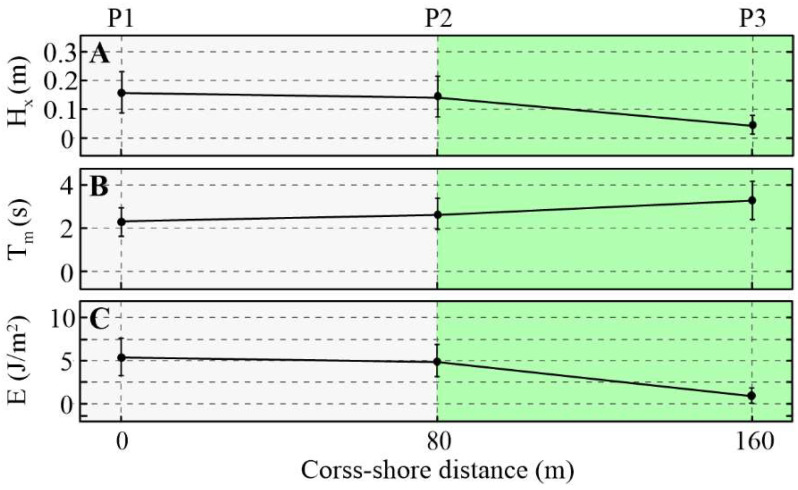
Wave parameter conditions along the transect. (**A**) Significant wave heights H_x_ (m); (**B**) mean wave periods Tm (s); and (**C**) total wave energy Em (J/m^2^).

Figure 5 presents the variation in the wave characteristics along the transect obtained from the wave energy density spectra for each data collection period. The mean significant wave height decreases from ~15.6 cm to ~14.9 cm and the total wave energy decreases from ~5.1 J/m^2^ to ~4.95 J/m^2^ from measurement station P1 to P2. Bottom friction is the main cause of wave energy dissipation on mudflats.

**Figure 6:**
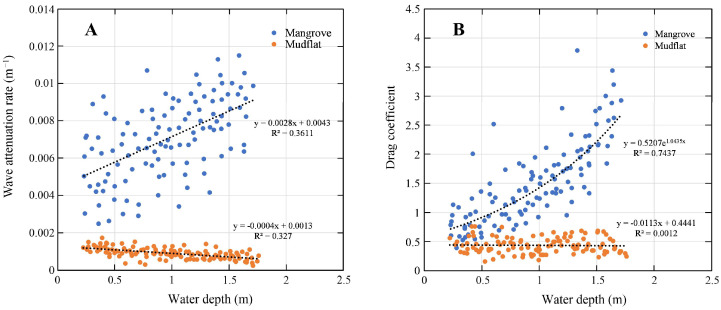
Variation in wave reduction (**A**) and drag coefficient (**B**) with water depth.

Figure 6A shows the measured wave height reduction per meter across the shore at the mudflat plotted against the local water depth at station P1. In the mudflat area, the rate of wave attenuation decreases with the increasing water depth from 0.0018 m^−1^ to 0.0005 m^−1^, and the linear regression of the wave height reduction per meter cross-shore versus the water depth is r = −4·10^−4^·h + 1.3·10^−3^ (R^2^ = 0.33). The drag coefficient (*C_d_*) varied between 0.1 and 0.9 and has very little relationship with the variation in water depth. A liner expression between the resistant coefficient and the water depth had a gain of −11.3 × 10^4^ (R^2^ = 0.0012).

### 2.4. Attenuation of Waves in the Mangroves

Typically, wave attenuation in the vicinity of mangroves is due to the bottom friction and drag force. Bottom friction is caused by bed roughness. Drag force, on the other hand, is usually associated with the resistance exerted by mangrove structures such as trunks, roots and canopies (in cases where mangroves are completely submerged). As shown in Figure 5, the mean significant wave height gradually decreased from ~14.9 cm at the forest fringe (station P1) to ~5.6 cm at the back of the forest (station P3) (~−62%). Approximately 3.75 J/m^2^ (~76%) of wave energy is dissipated within 80 m of the mangrove. Both the reduction in wave height per meter across the shore and the attenuation of energy per meter were much higher in the mangrove compared to on the mudflat. This demonstrates that the contribution of mangrove vegetation as a friction factor is obvious. The obtained wave attenuation rates of mangroves were in the range of 0.002–0.012 m^−1^.

The linear regression relationship between wave height reduction per meter of cross-shore in the mangroves and water depth can be expressed as r = −2.8 × 10^3^·h + 4.3 × 10^3^ (R^2^ = 0.36) (Figure 6A). As expected from this relationship, the drag coefficient Cd also showed a positive correlation with water depth, satisfying the exponential relationship of r = 0.5207e^1.0435h^ (R^2^ = 0.74) (Figure 6B). The characteristics of these relationships reveal that as the water level increases, the resistance of the mangrove to waves comes not only from the trunk and roots but also from the canopy layer.

**Figure 7:**
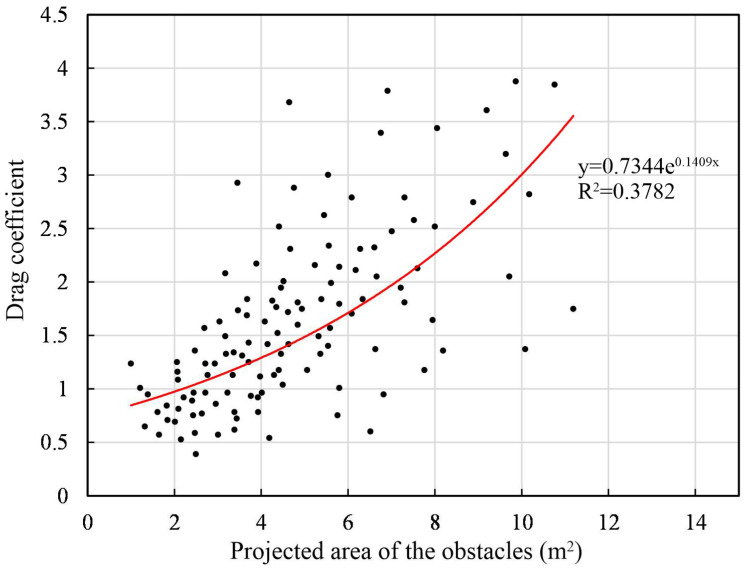
The relationship between the projected area of the obstacle per meter width and the drag coefficient.

Figure 7 illustrates the relationship between the drag coefficient *C_d_* and the projected area of the obstacle created by the vegetation in the direction of the incident wave *A_m_*, where *A_m_* is estimated from vegetation parameters such as the trunk height, trunk width, leaf height and leaf width of trees between hydrological observation sites [2]. *A_m_* increased significantly when the water level exceeded the canopy layer. Since the drag force was caused by the vegetation, *C_d_* showed a good positive correlation with *A_m_*. The exponential function of

(1) $$C_{d}=0.7344e^{0.1409A_{m}}$$

described this relation over the 80 m distance between station P2 and P3 with a correlation of 0.38.

**Figure 8:**
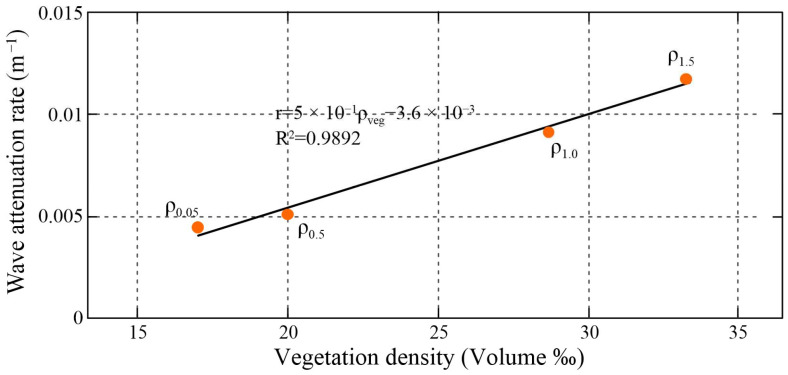
The correlation between the attenuation rate of wave height and the density of volumetric vegetation in a certain height above the forest surface. *ρ*_0.05_, *ρ*_0.5_, *ρ*_1.0_ and *ρ*_1.5_ denote the horizontal volumetric density of submerged vegetation at water depths of 0.05 m, 0.5 m, 1 m and 1.5 m, respectively.

Figure 8 presents the relationship between the wave attenuation rates and the volumetric vegetation density for a certain elevation range above the forest floor along the transect. It shows that the vegetation density within the forest zone increases with the water level, and the wave attenuation is rapidly dominated by the vegetation-induced drag force, which leads to a rapid increase in wave attenuation. The resulting best-fit expression for the wave attenuation rate to the vegetation density (*ρ_veg_*) in volume -‰ is obtained as follows:

This expression quantifies the trend of mangrove vegetation attenuation capacity

(2) $$r=5\times 10^{-4}\cdot \rho_{veg}-3.6\times 10^{-3}$$

with increasing vegetation density with water level rising along the transect. Roughly, within the mangroves, for every 10‰ increase in volumetric vegetation density, the generalized attenuation rate increases by approximately 0.005 m^−1^.

In addition, while the significant wave heights decreased along the transect, mean wave periods slightly increased towards the back of the forest. As shown in Figure 5A, the mean wave period changed from ~2.4 s at station P2 to ~3.1 s at station P3. This trend is also confirmed by the wave energy density spectrum presented in Figure 4, indicating that mangroves play a more effective role in attenuating the propagation of high-frequency waves (>0.3 Hz).

## 3. Discussion

### 3.1. Vegetation Features Affecting Wave Propagation in Mangroves

In a previous study, Brinkman [29] observed a reduction in total wave energy and significant wave height of approximately 95% and 75%, respectively, along a 40 m transect at the Oonoonba mangrove site in Australia. This value is much higher than that obtained in the present work. Nevertheless, the obtained wave attenuation rates (r = 0.002–0.012 m^−1^; Figure 6) compare well with previous observations such as at the Vinh Quang coast, Vietnam: 0.001–0.004 m^−1^ [27]; the coast of the Red River Delta: 0.004–0.012 m^−1^ [6]; and the west coast of southern Thailand: 0.002–0.012 m^−1^ [5]. The greater the density of distribution in the horizontal direction, the better the wave dissipation effect of vegetation. However, it has also been pointed out that when the vegetation density is so large that the vegetation spacing is smaller than the length of the vegetation-induced wake zone or overlaps with it, the change in the wave dissipation effect of vegetation will no longer be obvious [30], which means that there is a threshold value for the effect of horizontal distribution density on vegetation wave dissipation.

In addition, the variation in vegetation density is also reflected in the vertical direction and influenced by the water depth. The present study indicated the effect of *A. marina*—induced drag force on wave reduction increases considerably with increases in the water level (Figure 6 and Figure 8). The higher the water level, the more the vegetation inundation and the higher the resistance caused. However, the relationship between wave attenuation capacity and water depth can also change depending on the species. For example, *Kandelia candel* (L.) Druce, with its simple vertical structure and no roots emerging above the ground, causes a fairly constant wave attenuation with water depth [2]. *Rhizophora apiculata* Blume, on the other hand, showed a significant decrease in wave attenuation with increasing water depth because its canopy was located at a higher position and the shape of the pneumatophores tapers off upward [5]. Thus, obtaining detailed information on vegetation characteristics and hydrodynamic conditions is essential to understand the mechanism of vegetation-induced wave attenuation in mangroves.

Drag coefficient is a key parameter for quantifying the vegetation-induced dissipation. When calculating the drag coefficient using Equation (2), the effect of the resistance of mangrove trees with vertical configuration is presumed to be bottom friction [2]. However, as mentioned previously, mangrove trees can be stratified vertically, and each layer may have a different drag coefficient. Therefore, the drag coefficient *C_d_* calculated by Equation (2) is an average value, representing the overall drag of the mangroves. To obtain more accurate drag coefficients, new ideas and methods suitable for vertical structural changes need to be explored based on further observations and experiments.

### 3.2. Physical Processes Affecting Wave Propagation in Mangroves

During the cross-shore propagation of waves, the forest fringe and the sudden changes in vegetation density within the forest zone may induce local increases in wave height due to wave reflection [1,31]. This phenomenon was noted in some of the data collected at the forest fringe. A slight increase in wave height was observed at measurement site P1 relative to at site P2. However, no local increase in wave height due to wave reflection was observed within the mangrove forest. This should be attributed to the fact that the cross-shore density of the vegetation in the study area, as described above, exhibits a gradual increase from the forest fringe to the back of the forest without abrupt changes. Moreover, waves propagating in mangroves may also be refracted, bypassed and diffracted by vegetation, resulting in complex nonlinear wave–wave interactions, which in turn affect wave energy conversion. The slight shift of the spectral peaks of wave energy to lower frequencies in Figure 4 is probably the result of this effect. As for the influence of wave refraction, bypassing and diffraction and the resulting wave–wave interaction on the local wave height, unfortunately, the available data are insufficient to provide an explanation, which requires further targeted investigation and discussion.

It has been found that nearshore muddy sediments respond elastically to the pressure of advancing waves, resulting in a loss of wave volume [32]. In the study area, the seabed is mainly composed of muddy sediments. The muddy surface under the mangroves is subjected to relatively rapid consolidation due to water extraction and the growth of the underground roots of the mangroves, so the effect on hydrodynamic processes may still be significant. However, the effects of wave interactions with soft bottom sediments were not measured in this study and could not be included in the analysis of the data collected so far.

Wave shallowing also causes an increase in wave height in shallow coastal waters [33]. The rate of wave height change due to shallowing can be obtained by calculating the shallowing coefficient from the observed water depth and wave characteristic data based on the assumption of linear wave theory and then subtracting the shallowing coefficient from the unit value [5]. Accordingly, the wave height increase due to shallowing in the study area was calculated to be about 23% of the measured wave reduction in the mangroves. Consequently, the total attenuation rate of wave height by mangrove vegetation is expected to be greater than the presented (total) wave attenuation rate.

In general, processes such as vegetation-induced drag, bottom friction and shoaling in the mangrove zone all contribute to wave attenuation. However, isolating and quantifying the exact contribution of these processes to wave attenuation is not possible based on the currently available field data. Judging from the fact that the attenuation rate is minimal on the bare mudflats and significantly increases inside the mangrove vegetation (Figure 6), the dissipation of wave energy in mangroves is mainly accomplished due to turbulence caused by the vegetation. Therefore, the present observations are overall able to reflect the contribution of mangrove ecosystems to wave attenuation.

### 3.3. Quantifying Bio-Physical Interactions in Mangroves

As discussed above, the wave dissipation effectiveness of mangroves is the result of the interaction between mangrove characteristics (including tree vertical configuration and density) and physical factors (including wave characteristics, bed characteristics, slope, etc.). Understanding the contribution of any of these processes is key to future investigations of the capabilities and limitations of mangroves to attenuate wave energy and to determine thresholds for mangrove hydrodynamic habitats. Currently, there are three main methods to study wave attenuation caused by mangroves: field measurements, physical experiments and numerical simulations. Field measurements are the most straightforward and pragmatic method to examine the wave attenuation effects of mangrove vegetation, but to further study the bio-physical interactions of mangroves and to quantify the contribution of specific mangrove features to their attenuation capacity, mechanistic studies through physical experiments and numerical simulations are required. In contrast to field measurements, physical experiments and numerical simulations allow controlled observation and simulation of those processes that contribute to the total wave attenuation rate in mangroves: wave, vegetation-induced drag, bottom friction, viscous dissipation and shoaling. To our knowledge, few mechanistic studies have been carried out for this mangrove domain due to the lack of measured data. Accordingly, the detailed and integrated in situ observations of the vegetation, hydrodynamics and morphology of mangrove systems presented in this paper will provide important input for further mechanistic studies.

## 4. Materials and Methods

### 4.1. Study Site

Zhanjiang Bay (ZB) is a semi-enclosed shallow bay located on the eastern side of the Leizhou Peninsula in China, with its mouth facing southeast to the South China Sea (Figure 1A,B). Influenced by the tropical monsoon climate and oceanic climate, the annual average temperature of the area is approximately 23.4 °C and the annual average precipitation is approximately 1618.5 mm [34]. Rainfall is mainly concentrated in the months of April to September, which is also the season of typhoon storms. The tides in the bay are subject to an irregular semi-diurnal with a range of 2–3 m. Wind waves are the main source of wave energy, accounting for approximately 80% of the annual total [35].

The mangrove belt is located in the intertidal zone on the north coast of ZB (Figure 1C), with a near N-W direction and an area of about 0.5 km^2^, and it is part of the Zhanjiang National Mangrove Nature Reserve. *A. marina* is the most dominant mangrove species in this region. Given that the bottom slope of the coast is around 0.0037, large areas of mudflats in front of the mangroves dry up at low tide and can reach several hundred meters offshore at spring tide. The weather conditions and flat intertidal zone are favorable for mangrove growth.

### 4.2. Data Collection

To investigate the effectiveness of *A. marina* in wave attenuation, a cross-shore transect was designed that aligned with the prevailing direction of wave propagation in the shore and extended from the mudflat into the mangroves (Figure 1C,D). Three measurement stations were equidistantly positioned (~80 m) along the transect, of which station P1 was on the mudflat, station P2 on the fringe of the mangroves and station P3 in the mangrove forest. Since the seaward fringe of the mangroves is approximately the position of the mean sea level, the deployed measurement point P3 will not experience the whole tidal variation. Therefore, the observations were chosen to be conducted during the spring tide, which started on 22 May at 16:00 and ended on 29 May at 16:00, 2024. Moreover, a vegetation characterization survey quadrant was set up between stations P2 and P3 (Figure 1D). The size of the quadrant is 20 × 20 m^2^. The roots, stems, branches and leaves of the vegetation in the quadrant were quantified in detail and served as the basis for the vegetation cover of the wave transect. Wave measurements were performed by high-frequency pressure sensors. In order to obtain effective wave height data for shallow water depths, the instruments were buried with the sensors leveled at about 5 cm above the bed. The sampling frequency of the pressure sensors was set to 2 Hz, with burst lengths of 1200 samples at intervals of 30 min.

### 4.3. Data Processing

For the vegetation data, referring to Horstman et al. [5] and Best et al. [24], they were first transformed into the total horizontal coverage of vegetation elements within the quadrant at different levels above the bed, and then the volume of vegetation within a water column of arbitrary depth was calculated by integrating the horizontal vegetation coverage over the depth, and finally the ratio of the relative vegetation volume (volume-‰) to the total submerged volume was expressed as the vegetation density.

For wave data, the wave signal was first spectrally analyzed using a Fourier analysis scheme [36] to obtain the wave energy density spectra for each burst. Then, the total significant wave height, mean wave period and total wave energy for each burst were derived from the energy density spectra. Finally, data bursts were selected for time spans in which the entire transect was submerged, and the swell and infragravity wave bands were selected using a bandpass filter to determine their presence and magnitude within the mangroves, similarly to the method utilized by [24].

The wave attenuation rate was obtained from the following equation [27]:

(3) $$r=\frac{H_{x_{1}}-H_{x_{2}}}{H_{x_{1}}}\frac{1}{∆x}$$

where $H_{x_{1}}$ is the wave height at a seaside station *x*_1_, $H_{x_{2}}$ is the wave height at an inshore station *x*_2_ and Δ*x* is the distance between *x*_1_ and *x*_2_. The drag coefficient *C_d_*, a dimensionless number used to characterize the resistance generated by a fluid flowing over the surface of an object, is approximated as follows [2]:

(4) $$C_{d}=\frac{32\sqrt{2}}{\pi }\frac{h^{2}}{H_{x_{1}}∆x}\left(\frac{H_{x_{1}}}{H_{x_{2}}}-1\right)$$

where *h* is the water depth.

## 5. Conclusions

To investigate the wave attenuation effect of *A. marina* on the north coast of Zhanjiang Bay in southern China, cross-shore in situ observations were constructed to obtain the vegetation characteristics and wave variation along the mangrove belt. Based on the observation data, the variation in the friction coefficient caused by mangroves and its relationship with water depth and wave height were analyzed, and the wave attenuation ability caused by mangrove vegetation characteristics was discussed. 

It is found that energy losses due to bottom friction and viscous dissipation caused significantly lower attenuation rates on the bare mudflats than within mangrove vegetation. The observed mean significant wave height (on average) decreased by ~62% and wave energy decreased by ~76% over an 80 m long cross-shore section due to various bio-physical interactions in the mangrove ecosystem. Wave attenuation was most effective for high-frequency waves (>0.3 Hz). It is also found that the wave attenuation is positively correlated with the water depth and incident wave height. As the water depth/incident wave height increases during wave propagation, the waves are not only affected by the root and trunk parts but also damped by the canopy layer, resulting in faster attenuation. The drag force within the forest zone can be approximated by the function $C_{d}=0.7344e^{0.1409A_{m}}$, where *A_m_* is the projected area of the underwater obstacle at a certain water depth. The best-fit expression for the wave attenuation rate versus the vegetation density (*ρ_veg_*) in volume -‰ is $r=5\cdot 10^{-4}\cdot \rho_{veg}-3.6\cdot 10^{-3}$.

These findings emphasize the wave attenuation function of *A. marina* and accumulate quantitative information on the wave attenuation effect of mangroves, and they also provide basic data for modeling studies to investigate the processes contributing to the attenuation capacity of mangroves.

## Data Availability

The raw data supporting the conclusions of this article will be made available by the authors on request.

## References

[1] Massel S.R., Furukawa K., Brinkman R.M. (1999). Surface wave propagation in mangrove forests. Fluid Dyn. Res..

[2] Mazda Y., Magi M., Kogo M., Hong P.N. (1997). Mangroves as a coastal protection from waves in the Tong King delta, Vietnam. Mangroves Salt Marshes.

[3] Méndez F.J., Losada I.J. (2004). Anempirical model to estimate the propagation of random breaking and nonbreaking waves over vegetation fields. Coast. Eng..

[4] Cao H., Chen Y., Tian Y., Feng W. (2016). Field Investigation into Wave Attenuation in the Mangrove Environment of the South China Sea Coast. J. Coastal Res..

[5] Horstman E.M., Dohmen-Janssen C.M., Narra P.M.F., van den Berga F., Siemerink M., Hulscher S.J.M.H. (2014). Wave attenuation in mangroves: A quantitative approach to field observations. Coastal Eng..

[6] Quartel S., Kroon A., Augustinus P.G.E.F., van Santen P., Tri N.H. (2007). Wave attenuation in coastal mangroves in the Red River Delta, Vietnam. J. Asian Earth Sci..

[7] Zhou X., Dai Z., Pang W., Wang J., Long C. (2022). Wave attenuation over mangroves in the Nanliu Delta, China. Front. Mar. Sci..

[8] Hashim A.M., Catherine S.M.P. (2013). A laboratory study on wave reduction by mangrove forests. APCBEE Procedia.

[9] Husrin S., Strusinska A., Oumeraci H. (2012). Experimental study on tsunami attenuation by mangrove forest. Earth Planets Space.

[10] Maza M., Lara J.L., Losada I.J. (2019). Experimental analysis of wave attenuation and drag forces in a realistic fringe Rhizophora mangrove forest. Adv. Water Resour..

[11] Wang Y., Yin Z., Liu Y. (2022). Laboratory study on the drag coefficient for mangrove forests in regular waves. Ocean Eng..

[12] Cuc N.T.K., Suzuki T., de Ruyter van Steveninck E.D., Hai H. (2015). Modelling the impacts of mangrove vegetation structure on wave dissipation in Ben Tre Province, Vietnam, under different climate change scenarios. J. Coastal Res..

[13] Magdalena I., Kusnowo V., Azis I.M., Widowati (2021). 1D-2D Numerical Model for Wave Attenuation by Mangroves as a Porous Structure. Computation.

[14] Phan K.L., Stive M.J.F., Zijlema M., Truong H.S., Araninkhof S.G.J. (2019). The effects of wave non-linearity on wave attenuation by vegetation. Coast. Eng..

[15] Zhang Y., Yang Y., Yang K., Tan X., Sun X., Leng B., Zhou C., Zhu B. (2020). Non-linear wave attenuation quantification model improves the estimation of wave attenuation efficiency of mangroves. Estuar. Coast. Shelf Sci..

[16] FAO (Food and Agricultural Organization) (2007). The World’s Mangroves 1980-2005 (No. 153 FAO Forestry Paper).

[17] Giri C., Ochieng E., Tieszen L.L., Zhu Z., Singh A., Loveland T., Masek J., Duke N. (2011). Status and distribution of mangrove forests of the world using earth observation satellite data. Glob. Ecol. Biogeogr..

[18] Spalding M., Kainuma M., Collins L. (2010). World Atlas of Mangroves.

[19] Borsje B.W., van Wesenbeeck B.K., Dekker F., Paalvast P., Bouma T.J., van Katwijk M.M., de Vries M.B. (2011). How ecological engineering can serve in coastal protection. Ecol. Eng..

[20] Blankespoor B., Dasgupta S., Lange G.-M. (2017). Mangroves as a protection from storm surges in a changing climate. Ambio.

[21] Masson-Delmotte V., Zhai P., Pirani A., Connors S.L., Péan C., Berger S., Caud N., Chen Y., Goldfarb L., Gomis M.I. (2021). Climate Change 2021: The Physical Science Basis, Contribution of Working Group I to the Sixth Assessment Report of the Intergovernmental Panel on Climate Change.

[22] Dalrymple R.A., Kirby J.T., Hwang P.A. (1984). Wave diffraction due to areas of energy dissipation. J. Waterw. Port Coast. Ocean Eng..

[23] Ozeren Y., Wren D.G., Wu W. (2014). Experimental Investigation of Wave Attenuation Through Model and Live Vegetation. J. Waterw. Port Coast. Ocean Eng..

[24] Best Ü.S.N., van der Wegen M., Dijkstra J., Reyns J., van Prooijen B.C., Roelvink D. (2022). Wave attenuation potential, sediment properties and mangrove growth dynamics data over Guyana’s intertidal mudflats: Assessing the potential of mangrove restoration works. Earth Syst. Sci. Data.

[25] Quynh V.V. Effects on wave break, sea dike protection of the tree planting formula in the coastal salinity inundated areas. Proceedings of the Workshop on the Effect of Vegetation on Dike Design.

[26] Wolanski E., Mazda Y., Furukawa K., Ridd P., Kitheka J., Spagnol S., Stieglitz T., Wolanski E. (2001). Water circulation in mangroves, and its implications for biodiversity. Oceanographic Processes of Coral Reefs.

[27] Mazda Y., Magi M., Ikeda Y., Kurokawa T., Asano T. (2006). Wave reduction in a mangrove forest dominated by *Sonneratia* sp.. Wetlands Ecol. Manag..

[28] Battjes J.A., Stive M.J.F. (1985). Calibration and verification of a dissipation model for random breaking waves. J. Geophys. Res. Ocean..

[29] Brinkman R.M. (2006). Wave Attenuation in Mangrove Forests: An Investigation Through Field and Theoretical Studies. Ph.D. Thesis.

[30] Myrhaug D., Holmedal L.E., Ong M.C. (2009). Nonlinear random wave-induced drag force on a vegetation field. Coast. Eng..

[31] Méndez F.J., Losada I.J., Losada M.A. (1999). Hydrodynamics induced by wind waves in a vegetation field. J. Geophys. Res. Ocean..

[32] Gratiot N., Bildstein A., Anh T.T., Thoss H., Denis H., Michallet H., Apel H. (2017). Sediment flocculation in the Mekong River estuary, Vietnam, an important driver of geomorphological changes. C. R. Geosci..

[33] Van Rijn L.C. (2008). Principles of Fluid Flow and Surface Waves in Rivers, Estuaries, Seas and Oceans.

[34] Zhang Y., Li G. (2013). Analysis of port construction conditions in the Leizhou Bay. Ocean Eng..

[35] Zhao C. (1999). Hydrographic and sediment analysis of Zhanjiang Bay. J. Waterw. Harb..

[36] Hegge B.J., Masselink G. (1996). Spectral analysis of geomorphic time series: Auto-spectrum. Earth Surf. Process. Landf..

